# Applying collective motion models to study discordant individual behaviours within a school of fish

**DOI:** 10.1098/rsos.231618

**Published:** 2023-12-06

**Authors:** Andrea Trucco

**Affiliations:** ^1^ Department of Electrical, Electronic, Telecommunications Engineering, and Naval Architecture (DITEN), University of Genoa, 16145 Genoa, Italy; ^2^ Consorzio Nazionale Interuniversitario per le Telecomunicazioni (CNIT), 16145 Genoa, Italy

**Keywords:** school of fish, self-propelled particle model, collective motion, milling, individual emergent behaviour, counter-rotation

## Abstract

Computational models of collective motion successfully reproduce the most common behaviours of a school of fish, using only a few elementary interactions between individuals. However, their ability to also reproduce individual behaviours that are discordant from those of the group has not yet been adequately investigated. In this paper, a self-propelled particle model using three interaction zones is considered in relation to the counter-rotation of an individual: a phenomenon observable in real schools of fish milling in a torus, when an individual moves in the same torus but in the opposite direction for a certain period of time. This study shows that the interactions of repulsion, orientation and attraction between individuals moving at constant speed in a three-dimensional space, with asynchronous updating, can generate temporary counter-rotations. The analysis of such events sheds light on the mechanisms that start the counter-rotation and those that end it. Although the contribution of the repulsion interaction is often significant to start and terminate the counter-rotation, it does not prove to be decisive. Indeed, it is observed that even when interactions between individuals are limited to attraction alone, temporary counter-rotations of individuals occur, provided the fish density along the circumference is not uniform. Some of these conclusions, deduced from the simulations performed, are visually consistent with what is observed in some underwater video recordings of milling schools of fish.

## 1. Introduction

The collective motion of schools of fish, like more generally that of animal groups [1,2], has attracted considerable attention in the scientific community and is still being studied today [3–6]. Some models have been proposed that, by defining a set of interactions between the individuals comprising a school of fish, we are able to reproduce the collective behaviours of schools of fish that are most frequently observed in nature. Such models are commonly referred to as individual-based or agent-based models [5,7–9]: in them, the evolution of position and velocity of each individual is governed by a set of differential equations [10–17] or a set of difference equations [4,18–30] at discrete instants of time. Despite the simplicity of the interactions included in these models, they have been successful in replicating collective behaviours with good accuracy.

Over the years, it has been observed that models including different sets of interactions or governing the same interactions with different rules can reproduce the same collective behaviours [7,9]. While this fact does not help to clarify which interactions are adopted by real fish, it shows that schools of fish can achieve a given behaviour by deploying different rules of social interactions.

In 2002 Couzin *et al*. [18], elaborating on the approach previously introduced by Aoki [31] and Reynolds [32], refined and proposed one of the most successful models in the scientific community: by adjusting a few parameters, this self-propelled particle model [2] is able to reproduce the most frequent collective motion patterns (namely, milling, swarm and parallel aligned motion). For this reason and because of its simplicity, this model has been the subject of numerous analyses, refinements and comparisons, and is still widely used [3,24,25,28,29,33–35]. In its original version, the model allows each fish in the school, proceeding at a constant speed, to vary its direction on the basis of three local interactions: repulsion, orientation and attraction, with respect to its neighbours contained in three spheres centred on the fish itself and of increasing radius. The model also predicts that the fish has a limited field of view, a maximum turn rate and is subject to random errors in aligning itself to a new direction. For the sake of brevity, this model will hereafter simply be called the *three-zone model* [7].

Many experimental investigations have been performed to confirm the interactions assumed in aprioristic models or to decipher the interactions actually present in mobile animal groups [6,15,19,36–43]. Some of these investigations have shown that in certain groups of birds or fish [38–40] the orientation interaction does not seem to be present. Indeed, there are models in which the collective behaviours already mentioned emerge even in the absence of orientation [11,12,14,16,44] or in the presence of attraction alone [16,23,25,27]. Finally, it is worth noting that in many works, the investigation was performed by restricting the school of fish to moving in one plane [9–17,22,23,26,27,29,30,45] and setting precise initial conditions to start its movement [10,12,13,29,45].

The study of how to help collective behaviours and transitions from one behaviour to another emerge has left little room for the analysis of individual behaviours, which can sometimes be discordant, i.e. contrary to the dominant behaviour of the group. Although the impact of behavioural differences between individuals on the collective behaviour has been investigated (e.g. [1,22,44,46]), the attention given in the literature to discordant behaviours of a single individual or small groups, which emerge from a model in which all individuals have identical characteristics, has been rather limited so far [17,42,47,48]. This paper analyses the case of a fish that, while the school of fish mills within a torus in a certain direction, begins a mill within the torus occupied by the other individuals, but in the opposite direction to them. The behaviour referred to concerns only one or very few fish and lasts for a short period of time compared to the duration of the school milling. It is therefore a fish that, milling together with the others, at a given instant changes direction of milling, travels a fraction of the circumference (or a few turns of it) in the opposite direction, and then returns to mill in the dominant direction. In the following, this behaviour will be referred to as counter-rotating fish. The electronic supplementary material contains a list with links and comments to some underwater video recordings in which this individual counter-rotating behaviour can be observed in schools of sardinella, barracuda and jack fish.

A counter-rotating fish should not be confused with double milling, observed in nature and reproduced by some individual-based computational models [7,11,13,19,23,27,45], in which some fish travel around the circumference in one direction, while the rest travel in the opposite direction. The difference with the case examined in this paper is twofold: in double milling both directions of milling are travelled by many individuals and this behaviour remains stable for a long time, often for the entire duration of the torus. The double milling emerges by choosing parameter values in a certain region of the parameter space or by imposing particular initial conditions. This behaviour disappears in favour of single milling, in which all fish mill in the same direction, when, depending on the model: a strong orientation interaction or a spherical repulsion zone is introduced [7,10,11]; random initial conditions are imposed [13]; or a blind zone is introduced behind the individual [23].

The type of counter-rotation considered in this paper has rarely been shown or described in the literature. To the best of the author's knowledge, this has only occurred in papers addressing the mechanisms governing transitions of schools of fish from one collective motion pattern to another [17,42,47,48]. In particular, analyses of experimental observations of groups of golden shiners in a shallow tank [42] revealed that some individuals initiate a temporary counter-rotation when the rotating school encounters a tank wall and, because of this, it undergoes a transition from the milling state to the polar state. Moving from experimental observations to simulations, the electronic supplementary material videos in [47] show some counter-rotations of very short duration in schools of fish repeatedly transitioning from the milling state to the polar state and vice versa. However, the attention of these papers does not focus on counter-rotations as such, but focuses more generally on the mechanisms (including the discordant behaviours of a few individuals) involved in transitions between different collective motion patterns.

The first objective of this study was to investigate whether, by using the three-zone model (activating all the interactions originally predicted or activating the attraction alone) and setting the parameters to simulate a single milling school of fish, the discordant individual behaviour described above occurs. Secondly, an attempt was made to find an explanation for this behaviour, depending on the interactions activated. Finally, the possible relationships between the presence of counter-rotations and the transition of the school of fish from the milling state to another state of collective motion are studied. Quantification of the observed phenomena was provided by numerical investigation performed through many independent simulations for each parameter setting tested. The investigations mentioned introduce new scientific knowledge since the counter-rotating fish, in relation to computational models of collective motion, is a phenomenon that has not yet received specific attention, especially in the case of models working in three-dimensional space. More generally, this paper contributes to a field poorly explored to date, that of individual behaviours emerging from computational models of interaction between group individuals. An important aspect of this study is to contribute to formulating an answer to the question: are models born for collective behaviour also able to reproduce discordant individual behaviours and can they help to understand the underlying processes?

This paper is organized as follows. Section 2 describes the three-zone model (fitted with all interactions or with attraction only), the metrics adopted to assess collective and individual behaviours and the simulation set-up. Section 3 illustrates and quantifies the results obtained with the different versions of the model and in relation to counter-rotation starting and ending processes. Finally, §§4 and 5 present a discussion of the results obtained and the conclusions reached, respectively.

## 2. Methods

### 2.1. The three-zone model

According to experimental observations, Couzin *et al*. [18] proposed a self-propelled particle model in which each individual in a school of fish moves in a three-dimensional space with a velocity whose modulus remains constant and whose direction depends on interactions with its neighbours. Changes in direction cannot exceed the maximum turn rate of which the individual is capable and include random errors. While space is continuous, time is discretized at Δ*_t_* step. The school of fish is composed of *N* individuals, whose position at time *t* is indicated by the vector **r**_*i*_(*t*), *i* = 1, 2, …, N, and whose velocity is equal to $v_{i}(t)=v_{0}{\hat{v}}_{i}(t)$, where *v*_0_ is the constant modulus (also called speed) and ${\hat{v}}_{i}(t)$ is a unit vector indicating the direction. The maximum turn in the unit time of which the individual is capable is indicated by the angle *ρ*_max_.

The position of the *i*-th individual at instant *t* + Δ*_t_* is calculated as follows:

2.1 $$r_{i}(t+\Delta_{t})=r_{i}(t)+\Delta_{t}v_{i}(t+\Delta_{t})$$

where $v_{i}(t+\Delta_{t})=v_{0}{\hat{v}}_{i}(t+\Delta_{t})$ and the unit vector ${\hat{v}}_{i}(t+\Delta_{t})$ is calculated based on the presence of other individuals in three interaction regions. These are three concentric spheres, centred at $r_{i}(t)$, called zones of repulsion, orientation and attraction, whose radii are *R_r_*, *R_o_*, *R_a_*, *R_r_* < *R_o_* < *R_a_*, respectively. A blind zone is also defined, located behind the individual, within which the fish cannot detect the presence of other individuals. The blind zone is a right circular cone whose apex is located at $r_{i}(t)$, whose axis extends infinitely along the direction $-{\hat{v}}_{i}(t)$, whose aperture angle is 360°-*θ*, where *θ* represents the field of perception of the individual.

The set $S_{r}$ contains the indices *j*, *j* ≠ *i*, of all the individuals that, at time instant $\tilde{t}$, are contained in the repulsion zone of the *i*-th individual, whose number is equal to *n_r_*. The set $S_{o}$ contains the indices *j*, *j* ≠ *i*, of the individuals that, at time instant $\tilde{t}$, are: outside the repulsion zone, outside the blind zone and contained in the orientation zone, whose number is equal to *n_o_*. Finally, the set $S_{a}$ contains the indices *j*, *j* ≠ *i*, of individuals who, at time instant $\tilde{t}$, are: outside the orientation zone, outside the blind zone and contained in the attraction zone, whose number is equal to *n_a_*. In the original three-zone model, the time instant $\tilde{t}$ corresponds to *t* and the blind zone does not apply to repulsion, but only to orientation and attraction [28].

In terms of priority, the dominant interaction is that of repulsion. If *n_r_* > 0, the *i*-th individual will attempt to move away from all neighbours contained in the set $S_{r}$, choosing as desired direction that of the vector $d_{\mathrm{des}}(t+\Delta_{t})$ which, in this case, corresponds to the repulsion vector defined as follows:

2.2 $$d_{r}(t+\Delta_{t})\;=-\sum_{\;j\in S_{r}}N\{r_{j}(\tilde{t})-r_{i}(t)\}$$

and

2.3 $$d_{\mathrm{des}}(t+\Delta_{t})\;=d_{r}(t+\Delta_{t}),$$

where N{.} is the operator that normalizes the modulus of a vector, N{a}=a/|a|, where |a|  is the modulus of the vector **a**. The result of N{.} is therefore a unit vector.

If the repulsion zone does not contain any individuals (i.e. *n_r_* = 0), the desired direction for the *i*-th individual is set based on the individuals present in the orientation and attraction zones, if any. First, the orientation vector:

2.4 $$d_{o}(t+\Delta_{t})\;=\sum_{\;j\in S_{o}}{\hat{v}}_{j}(\tilde{t}),$$

and the attraction vector:

2.5 $$d_{a}(t+\Delta_{t})\;=\sum_{\;j\in S_{a}}N\{r_{j}(\tilde{t})-r_{i}(t)\},$$

must be calculated (if *n_o_* = 0 or *n_a_* = 0, the corresponding vector is null). The desired direction is then determined as follows:

2.6 $$d_{\mathrm{des}}(t+\Delta_{t})\;=\{\begin{array}{ccc} {\hat{v}}_{i}(t) & \mathrm{if} & n_{o}=n_{a}=0\\ d_{o}(t+\Delta_{t})\;+d_{a}(t+\Delta_{t}) & \mathrm{otherwise} & \end{array}.$$

To account for errors in the individual's perception and movement, the desired direction $d_{\mathrm{des}}(t+\Delta_{t})$ is rotated by a random angle taken from a spherical distribution characterized by zero mean and standard deviation *σ*. The desired direction perturbed by the random error is denoted by ${d^{\prime }}_{\mathrm{des}}(t+\Delta_{t})$, while the absolute value of the angle between ${d^{\prime }}_{\mathrm{des}}(t+\Delta_{t})$ and ${\hat{v}}_{i}(t)$ is denoted by *β*. The new direction of movement of the *i*-th individual is finally calculated as follows:

2.7 $${\hat{v}}_{i}(t+\Delta_{t})=\{\begin{array}{c} N\{{d^{\prime }}_{\mathrm{des}}(t+\Delta_{t})\;\}\;\;\;\;\;\;\;\;\;\;\;\;\;\;\;if\;\beta \leq \rho_{\max}\Delta_{t}\\ R\{{\hat{v}}_{i}(t),{d^{\prime }}_{\mathrm{des}}(t+\Delta_{t})\;\}\;\;\;\;\mathrm{otherwise}\;\;\;\;\;\;\; \end{array},$$

where R{a,b} is the operator that rotates the vector **a** towards the vector **b**, performing a rotation equal to *ρ*_max_Δ*_t_*.

In the original three-zone model, the update of the school of fish status is done synchronously [8], by calculating the new position $r_{i}(t+\Delta_{t})$ of each individual based on the positions and velocities of the other fish at the time instant *t*. This means that in the equations above $\tilde{t}=t$. It was later observed that asynchronous updating is a way to introduce a further stochasticity and to achieve a better fit to experimental observations [8,22,23,26,27,49]. Asynchronism is implemented by applying the same rules seen above, but choosing the individual to be updated in random order [8,26]. More in detail, to calculate the position and the speed of the *i*-th individual at the time instant *t* + Δ*_t_*, the positions and the speeds at the instant *t* of those fish that have yet to be updated are used, while for the fish that have already been updated, the positions and velocities at time *t* + Δ*_t_* are used. Therefore, $\tilde{t}=t$ if the *j*-th fish has not yet been updated, $\tilde{t}=t+\Delta_{t}$ if the *j*-th fish has already been updated. The update cycle ends when all the individuals in the school of fish have been updated following a random order that changes with each cycle.

Another aspect of considerable importance is the initial conditions in which the school of fish is set at the time instant the simulation starts [1]. To influence the emerging collective behaviour as little as possible, the *N* individuals are arranged in random positions within a sphere of radius *Y*, with initial directions also chosen randomly in the three-dimensional space [18,28].

### 2.2. Attraction-based model

The simplicity of the three-zone model coupled with its ability to help emerge, depending on the parameter setting, all the most common collective behaviours, have made it a very appreciated working tool and the subject of numerous modifications and refinements for a variety of purposes [3,24,25,28,29,33–35].

Some studies based on experimental observations of schools of fish [39,40] have questioned the orientation interaction, of which they found no evidence in the species examined. This has contributed to the development of models in which this interaction is not included. For this reason, in addition to the original three-zone model, this paper also considers the extreme case in which only one interaction is present, namely, the attraction interaction. Previously, Strömbom *et al*. [23,26,27] demonstrated the possibility of obtaining milling, swarm and parallel aligned motion, with a model similar to the three-zone model in which, however, repulsion and orientation are not present. Parameter adjustment and asynchronous updating were required.

Testing the effects of the attraction interaction alone can be done with the three-zone model described in the previous subsection, simply by setting the radius of the spherical zones of repulsion and orientation to zero (i.e. *R_r_* = *R_o_* = 0). In this way, all fish inside the sphere of attraction and outside the blind zone will contribute to the attraction vector $d_{a}(t+\Delta_{t})$ which, in turn, will coincide with the vector expressing the desired direction, $d_{\mathrm{des}}(t+\Delta_{t})$. The three-zone model modified in this way does not correspond exactly to any of the variants proposed by Strömbom *et al*. [23,26,27], but the main difference lies in the fact that in this study, the simulation takes place in three-dimensional space instead of being confined to a plane.

### 2.3. Metrics for collective and individual behaviours

Emergent collective behaviour can be effectively characterized through the polarization and rotation coefficients of the school of fish, both defined to be between 0 and 1 [18,28,42]. The polarization coefficient:

2.8 $$C_{p}(t)=\left|\frac{1}{N}\sum_{i=1}^{N}{\hat{v}}_{i}(t)\right|,$$

increases with the degree of alignment between the individuals in the school of fish, while the rotation coefficient requires defining the centroid of the school:

2.9 $$r_{c}(t)=\frac{1}{N}\sum_{i=1}^{N}r_{i}(t),$$

and the normalized sum, m(t), of the angular moments of the individuals, calculated using the unit vectors of the position relative to the centroid and velocity:

2.10 $$m(t)=\frac{1}{N}\sum_{i=1}^{N}N\{r_{i}(t)-r_{c}(t)\}\;\times \;{\hat{v}}_{i}(t).\;$$

The rotation coefficient is the modulus of this vector: $C_{r}(t)=|m(t)|$. It increases with the degree of milling of the school of fish around its centroid, until it reaches 1 for a school uniformly distributed along a circumference, whose fish all move in the same direction.

The rotation period of the school of fish can be estimated by measuring the angle of rotation, with respect to the centroid, that each individual travels in a time interval *T* and then averaging the results over all the individuals. After defining $r_{i}^{1}=N\{r_{i}(t-T/2)-r_{c}(t-T/2)\}$ and $r_{i}^{2}=N\{r_{i}(t+T/2)-r_{c}(t+T/2)\}$, the angle of rotation for the *i*-th fish can be computed as: $\psi_{i}(t)=\arccos(r_{i}^{1}\cdot r_{i}^{2})$. Consequently, the school rotation period at time *t*, *O*(*t*), will be:

2.11 $$O(t)=\frac{T}{N}\sum_{i=1}^{N}\frac{2\pi }{\psi_{i}(t)}\;\;.$$

Similarly, the average radius of the torus at time *t*, *R*_tor_(*t*), can be calculated as follows:

2.12 $$R_{\mathrm{tor}}(t)=\frac{1}{N}\sum_{i=1}^{N}|r_{i}(t)-r_{c}(t)|\;.$$

Assuming that a school of fish is milling around its centroid (i.e. *C_p_* close to 0 and *C_r_* close to 1), the counter-rotation coefficient for the *i*-th individual can be defined:

2.13 $$c_{i}(t)=m(t)\cdot [N\{r_{i}(t)-r_{c}(t)\}\times {\hat{v}}_{i}(t)],$$

in order to identify any fish milling in the opposite direction to the school. By construction, the coefficient *c_i_* is between –1 and 1: for *N* ≫ 1, *c_i_* tends to 1 if the *i*-th fish mills in the direction of the school, while it tends to −1 if the fish mills in the opposite direction. During the movement, the *i*-th fish will be subjected to repulsions that may contribute to keeping the current rotation or to changing its direction. If *n_r_* > 0 at time *t*, a modified version of the counter-rotation coefficient, called the repulsion coefficient, defined as follows:

2.14 $$p_{i}(t)=m(t)\cdot [N\{r_{i}(t)-r_{c}(t)\}\times N\{d_{r}(t+\Delta_{t})\}],$$

can be adopted to measure such a contribution. By construction, the coefficient *p_i_* is between −1 and 1: *p_i_* tends to 1 if the repulsion pushes the *i*-th fish to rotate in the same direction as the school, while it tends to −1 if the repulsion pushes the *i*-th fish to rotate in the opposite direction.

### 2.4. Simulation set-up

The simulations were performed assuming a school of fish consisting of *N* = 100 individuals, identical to each other, with a length equal to an arbitrary unit (called unit), free to move in an unbounded three-dimensional space. At the initial time instant, they are at random positions within a sphere with radius *Y* = 10 units and are oriented in random directions. In the simulations using the three-zone model, the other parameters were set to obtain a milling group with high probability, as discussed in [18]. Table 1 lists the values initially assigned to the model parameters and shows the variation intervals subsequently considered to study the impact of *N* and *θ* on the phenomena observed. The random perturbation of the desired direction was cancelled out, by setting *σ* = 0. The reason for this is that, on the one hand, asynchronous updating already introduces a significant degree of stochasticity, and on the other hand, the absence of random errors helps to identify the reasons why an individual is rotating against the rest of the school of fish.

**Table 1.** Values of the parameters initially used in the three-zone model, when the repulsion, orientation and attraction interactions are all active. (The intervals used subsequently to investigate the impact of *N* and *θ* are in square brackets.)

parameter	symbol	unit of measurement	value
number of individuals	*N*	—	100; [50, 200]
time step	Δ*_t_*	s	0.1
speed	*v*_0_	unit s^−1^	3
zone of repulsion	*R_r_*	unit	1
zone of orientation	*R_o_*	unit	3.5
zone of attraction	*R_a_*	unit	16
field of perception	*θ*	degree	270; [225, 295]
max. turn rate	*ρ*_max_	degree s^−1^	40
error standard dev.	*σ*	degree	0

Each simulation has a duration of 2500 time steps. According to [9,42], the school of fish is considered to be in: the milling state when the polarization coefficient, *C_p_*(*t*), is less than 0.35 and the rotation coefficient, *C_r_*(*t*), is greater than 0.65; the polar state (i.e. the parallel aligned motion) when *C_p_*(*t*) is greater than 0.65 and *C_r_*(*t*) is less than 0.35; the swarm state when both *C_p_*(*t*) and *C_r_*(*t*) are less than 0.35; or the transitional state outside these ranges. When the school of fish is considered to be in the milling state, the counter-rotation coefficient of each individual is measured: the *i*-th individual is considered to be in a counter-rotation state if it maintains *c_i_*(*t*) < −0.3 for a period of at least 30 time steps. To avoid excessive fragmentation of counter-rotation occurrences, if two intervals in which *c_i_*(*t*) is less than −0.3 are separated by a number of time steps less than or equal to 10, they are considered as a single interval that satisfies the counter-rotation condition.

Moving from the three-zone model to the attraction-based model, the identification of the parameters that most likely lead the school of fish into milling is more critical. Indeed, the absence of the orientation interaction means that double-milling behaviour (as an alternative to swarm and polar motion) emerges more easily [23]. However, a careful exploration of the parameter space has shown that the values reported in table 2 are most likely to generate the milling of the school of fish in one direction, with no individuals remaining stably in the counter-rotation state. Further details are provided later.

**Table 2.** Values of the parameters used in the three-zone model, when only the attraction interaction is active.

parameter	symbol	unit of measurement	value
number of individuals	*N*	—	100
time step	Δ*_t_*	s	0.1
speed	*v*_0_	unit s^−1^	3
zone of repulsion	*R_r_*	unit	0
zone of orientation	*R_o_*	unit	0
zone of attraction	*R_a_*	unit	16
field of perception	*θ*	degree	290
max. turn rate	*ρ*_max_	degree s^−1^	65
error standard dev.	*σ*	degree	0

## 3. Results

### 3.1. Counter-rotation with significant contribution of repulsion

Using the set-up described above and the parameters in table 1, 100 simulations were performed, each lasting a total of 2500 time steps, corresponding to 4 min and 10 s. In these simulations, the school of fish maintains the milling state for a high fraction of time steps and, in each of them, a few individuals were detected to have entered the counter-rotation state, subsequently returning to milling in the direction of the school. However, before providing a detailed analysis regarding the simulations as a whole, it is preferable to observe the ongoing phenomena by focusing on some specific simulations. The significance of these phenomena and the underlying mechanisms will be verified and quantified through the numerical investigation described in a subsequent subsection.

**Figure 1.**
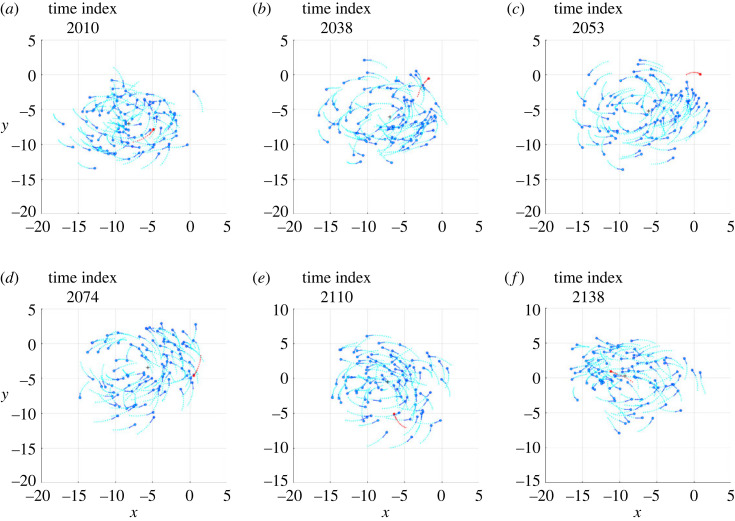
Six frames taken from the Sim*α* simulation. The circles and the segments following them (with respect to the direction of motion) indicate the current positions and directions of all individuals, respectively. The dots, on the other hand, show the positions occupied by each individual in the 11 previous time instants. The black cross is the current position of the centroid. The individual performing a temporary counter-rotation is the only one in red. (*a*) The individual mills in the same direction as the others. (*b*) After passing close to the centroid, the individual crosses the trajectories of the others and moves to the outside of the torus in which the school of fish mills. (*c*) The individual begins the milling in the opposite direction to the others. (*d*) The counter-rotation is in progress. (*e*) The individual in counter-rotation moves towards the centroid of the school of fish. (*f*) After passing the centroid, the individual resumes milling in the same direction as the school.

**Figure 2.**
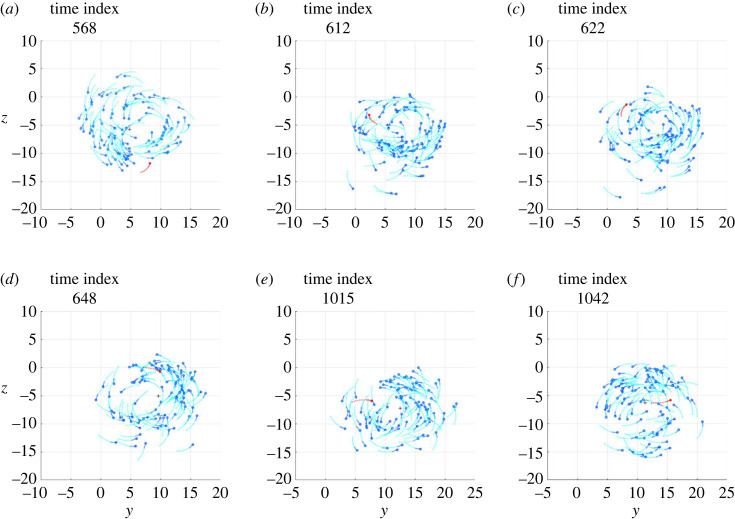
Six frames taken from the Sim*β* simulation, the meaning of which is described in figure 1 caption, as well as the graphic conventions adopted.

The electronic supplementary material, videos S1 and S2 show, in three-dimensional space, the clips of two simulations, denoted Sim*α* and Sim*β*, in which an individual, during the milling of the school of fish, undertakes a counter-rotation, maintains it for a certain period and, finally, returns to mill in the same direction as the other fish. In the Sim*α* case, the individual undertakes a counter-rotation that lasts less than a full turn, while in the Sim*β* case the individual undertakes a counter-rotation that lasts a few turns, before realigning with the school of fish. The projection of the schools of fish shown in S1 and S2 on the most indicated plane among those containing the Cartesian axes is visualized in the electronic supplementary material, videos S3 and S4, respectively. From these videos, six frames were isolated to illustrate the salient phases of the phenomenon, visible in figures 1 and 2, respectively.

**Figure 3.**
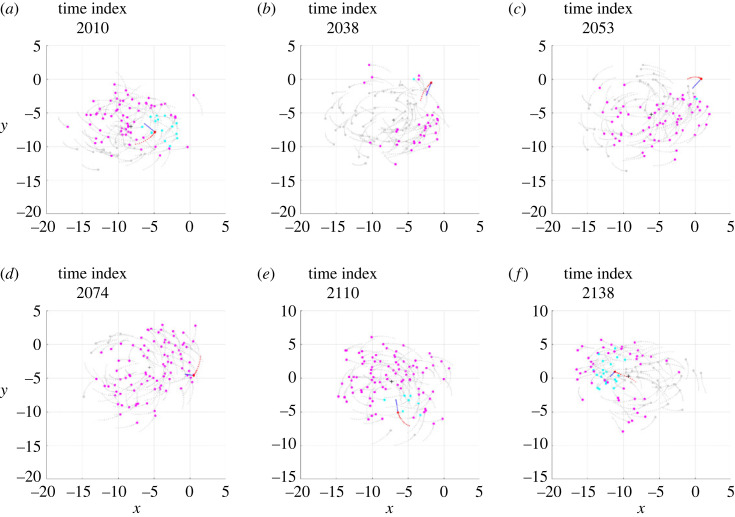
Six frames from the Sim*α* simulation, the meaning of which is described in figure 1 caption. In this analysis the individual performing a temporary counter-rotation is in red; a blue vector of constant modulus is applied to it, indicating the direction of the vector $d_{\mathrm{des}}(t+\Delta_{t})$; the pink circles are the individuals exerting attraction, contained in the set $S_{a}$; the blue circles are the individuals exerting orientation, contained in the set $S_{o}$; the grey circles are the individuals exerting no interaction.

**Figure 4.**
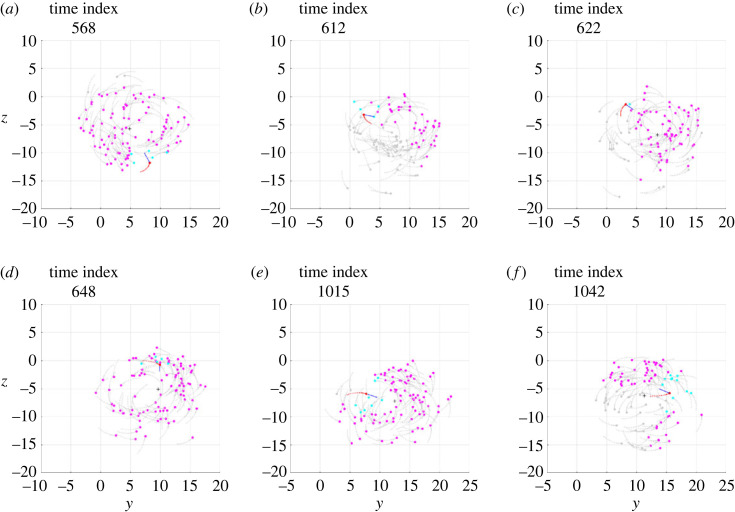
Six frames from the Sim*β* simulation, the meaning of which is described in figure 1 caption. In this analysis the individual performing a temporary counter-rotation is in red; a blue vector of constant modulus is applied to it, indicating the direction of the vector $d_{\mathrm{des}}(t+\Delta_{t})$; the pink circles are the individuals exerting attraction, contained in the set $S_{a}$; the blue circles are the individuals exerting orientation, contained in the set $S_{o}$; the grey circles are the individuals exerting no interaction.

In order to analyse the interactions that cause the individual to mill in the opposite direction, the Sim*α* and Sim*β* simulations were studied by highlighting which individuals within the repulsion, orientation and attraction zones, at each time step, contribute to defining the desired direction $d_{\mathrm{des}}(t+\Delta_{t})$ for the individual under investigation. Electronic supplementary material, videos S5 and S6 show the results of this analysis and contain a vector of constant modulus that, at each time step, shows the direction of the vector $d_{\mathrm{des}}(t+\Delta_{t})$. Figures 3 and 4 show the same results, limited to the six salient frames, the same as those already used for figures 1 and 2.

First of all, it should be noted that the density of fish in the toroidal space they travel through is not uniform: although there are no parts of the torus totally devoid of fish, in both simulations a large group of fish is observed to mill closer together than in the rest of the torus. Immediately before initiating the counter-rotation, the individual under investigation mills in the same direction as the school (*phase a*, figures 1*a* and 2*a*), close to the group with the highest density, with the vector of the desired direction pointing towards the centroid of the school (figures 3*a* and 4*a*). This pointing is usual for all individuals making a circular trajectory and is owing to the combined effect of neighbours exerting orientation and those, more numerous, exerting attraction.

**Figure 5.**
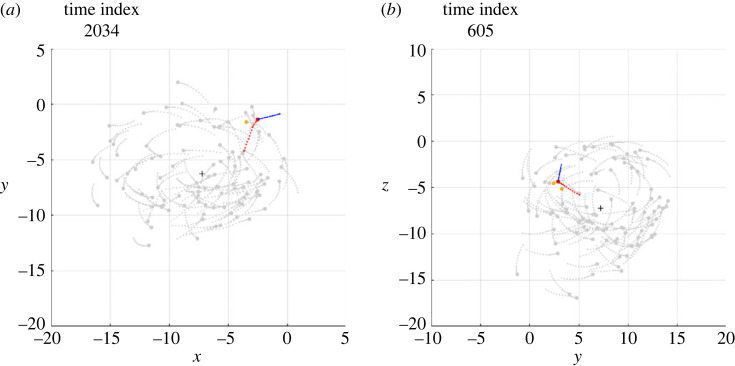
Examples of repulsion contributing to starting the counter-rotation of the individual in red. (*a*) Simulation Sim*α*. (*b*) Simulation Sim*β*. The frames in (*a*) and (*b*) are placed a few time steps before the frames shown in figures 3*b* and 4*b*, respectively. The graphic conventions are those described in figure 3 caption. In addition, the gold circles are the individuals exerting repulsion, contained in the set $S_{r}$.

After a passage near the centroid (passage of individuals near the centroid is commonly observed, both in simulations and in real schools of fish, without necessarily giving rise to counter-rotations), the individual crosses the trajectories of the fish in front of it, moving to the outer part of the torus (*phase b*, figures 1*b* and 2*b*). In performing this manoeuvre, in order to avoid collisions, the desired direction is governed several times by repulsion and contrary to the direction of the school milling, as can be seen in the electronic supplementary material, videos S5 and S6 and, partially, in figure 5. As a consequence, when the individual reaches the outer part of the torus, it is oriented in such a way as to be more attracted by the fish following it (in the direction of the school milling) than by those ahead of it (many of which are in the blind zone), as can be seen in figures 3*b* and 4*b*.

Outside the torus there are few orientation interactions (figures 3*c* and 4*c*): the attraction of the fish following the individual under investigation, not finding adequate opposition from the orientation, dominates and causes the individual to orientate in the opposite direction to its neighbours (*phase c*, figures 1*c* and 2*c*). As the desired direction returns to point towards the centroid of the school of fish (figures 3*c* and 4*c*), the counter-rotation begins. Although the orientation interaction, in general, causes the fish in the school to mill in the same direction, it is not strong enough to prevent the counter-rotating individual from continuing its trajectory for some time (*phase d*, figures 1*d* and 2*d*), keeping its desired direction pointing towards the centroid of the school (figures 3*d* and 4*d*).

**Figure 6.**
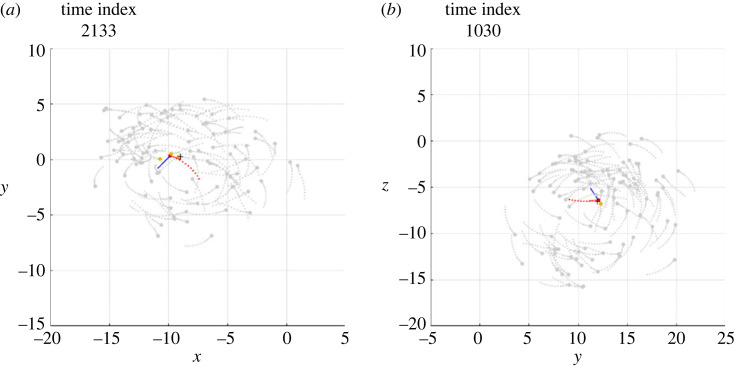
Examples of repulsion which helps the individual in red to resume milling in the same direction as the school of fish. (*a*) Simulation Sim*α*. (*b*) Simulation Sim*β*. The frames in (*a*) and (*b*) are placed a few time steps before the frames shown in figures 3*f* and 4*f*, respectively. The graphic conventions are those described in figure 3 caption. In addition, the gold circles are the individuals exerting repulsion, contained in the set $S_{r}$.

After a counter-rotation period of varying duration, the individual moves towards the centroid of the school of fish (*phase e*, figures 1*e* and 2*e*), aligning with its desired direction (figures 3*e* and 4*e*). In the vicinity of the centroid, to avoid collisions, the desired direction is repeatedly found to be governed by repulsion and aligned with the direction of the school of fish milling, as can be seen in the electronic supplementary material, videos S5 and S6 and, partially, in figure 6. These interactions orient the individual so that it is more attracted to the fish ahead of it (in the direction of the school milling) than to those following it. Added to this effect is the contribution of orientation, bringing the desired direction into alignment with the milling direction of the school of fish (figures 3*f* and 4*f*). The counter-rotation ends and the individual is disposed to mill in the same direction as the other fish (*phase f*, figures 1*f* and 2*f*).

### 3.2. Counter-rotation with reduced contribution of repulsion

The mechanism described in the previous subsection, using two examples, is the one that frequently characterizes the counter-rotations observed in the simulations performed, as will be demonstrated by the quantitative analysis in the next subsection. In such a mechanism, repulsion contributes significantly each time the individual changes direction of milling: either at the start of the counter-rotation, or at the resumption of milling in the school direction. In some cases, however, it has been observed that the change of direction occurs without the contribution of repulsion or, even, with an opposite contribution, which forces the individual to maintain the direction of milling already in place.

The electronic supplementary material, video S7 shows the projection on the plane of a simulation clip, indicated with Sim*γ*, in which a counter-rotation similar to those of the two examples described in the previous subsection is present. The analysis of the interactions taking place, visible in the electronic supplementary material, video S8, shows that in the phase that leads the individual to initiate the counter-rotation, numerous repulsions occur: for time indexes between 1935 and 1943 and between 1958 and 1964. Of these, only the one at time index 1958 pushes the individual towards counter-rotation, all the remaining ones contribute to maintaining the milling in the direction of the school of fish. Nevertheless, the individual undertakes the counter-rotation. The electronic supplementary material, videos S9 and S10 show a clip of a simulation, indicated by Sim*δ*, in which, just before the end of the counter-rotation, a number of repulsions occur (at time indexes 1375, 1376 and 1380): all of them facilitate the maintenance of the milling in the opposite direction to the school of fish. Nevertheless, the individual changes direction of milling and aligns with the milling of the school. Subsequently, the repulsions at time indexes 1384–1386 facilitate the individual's milling in the direction of the school of fish, but this occurs when the counter-rotation has already ended.

It can therefore be concluded that the repulsions, although present and significant in some cases (see the quantitative investigation in the next subsection), are not decisive either for the starting of the counter-rotation or for terminating it.

### 3.3. Quantitative analysis

The first investigation concerns the percentage of time steps the school of fish remains in the four different states. After analysing each simulation, the sample mean and sample standard deviation of these percentages are calculated using the 100 available simulations. The sample standard deviation is used to estimate the standard error of the mean (s.e.m.), which indicates the degree of reliability of the sample mean [50]. Specifically, the s.e.m. is equal to the sample standard deviation divided by the square root of the number of available values [50]. Since 100 simulations are always used in this work, the sample standard deviation is 10 times the s.e.m. In the remainder of the paper, these variables will be written as: sample mean ± s.e.m. [50]. The result is that the school of fish spends: 66% ± 1.2% of the time in the milling state; 8% ± 0.3% of the time in the swarm state; 26% ± 0.1% of the time in the transitional state. The polar state is never reached. These numbers capture the fact that random initial conditions cause the school of fish to start from the swarm state. It quickly moves into the transitional state and then reaches the milling state and remains there with good stability.

Focusing on the time steps in which the school of fish is in the milling state, the temporal average of the rotation period, *O*(*t*), and torus radius, *R*_tor_(*t*), can be computed for each simulation. The sample mean and s.e.m. of them over the 100 simulations are 5.773 ± 0.005 units for the radius and 16.31 ± 0.06 s (i.e. 163.1 ± 0.6 time steps) for the rotation period, showing that fluctuations among tori generated in successive simulations are small.

The main characteristics of the counter-rotations observed in the 100 simulations are summarized in table 3, where the following information is included: *n*^cnrt^, number of counter-rotations detected in one minute (i.e. 600 time steps) of milling state (sample mean ± s.e.m.); *o*^cnrt^, duration of a counter-rotation, measured in rotation cycles (sample mean ± s.e.m.); $C_{p}^{\mathrm{cnrt}},$ time average of the polarization coefficient of the school of fish, *C_p_*(*t*), during counter-rotation (sample mean ± s.e.m.); $C_{r}^{\mathrm{cnrt}},$ time average of the rotation coefficient of the school of fish, *C_r_*(*t*), during counter-rotation (sample mean ± s.e.m.); *P*_1_, counter-rotations for which, in the starting phase, the individual passes near the centroid (percentage); *P*_2_, counter-rotations for which, in the ending phase, the individual passes near the centroid (percentage); *P*_3_, counter-rotations for which, in the starting phase or in the ending phase or in both phases, the individual undergoes repulsions that significantly push it to start or end the counter-rotation, respectively (percentage).

**Table 3.** Characteristics of the counter-rotations observed in 100 simulations with the parameter setting of table 1 (*N* = 100, *θ*=270°). (See text for more details on definitions of characteristics.)

counter-rotation characteristic	symbol	value
number of occurrences per minute	*n*^cnrt^	1.19 ± 0.07
duration in rotation cycles	*o*^cnrt^	0.40 ± 0.02
polarization coefficient	$C_{p}^{\mathrm{cnrt}}$	0.26 ± 0.005
rotation coefficient	$C_{r}^{\mathrm{cnrt}}$	0.73 ± 0.003
passage near the centroid (start)	*P*_1_	91.7%
passage near the centroid (end)	*P*_2_	92.9%
significant repulsions (start OR end)	*P*_3_	63.7%

How the percentages *P*_1_, *P*_2_ and *P*_3_ are computed, exploiting *R*_tor_(*t*) and *p_i_*(*t*), is described in detail in appendix A.

Based on the values entered in table 3, it can be observed that:

(i) for every minute the school of fish maintains the milling state, approximately one counter-rotation occurs;
(ii) counter-rotations have on average a short duration, it being slightly less than half a rotation cycle;
(iii) the values of the rotation and polarization coefficients indicate that, during counter-rotations, the fish are non-uniformly distributed within the torus. These coefficients are considerably far from 1 and 0, respectively, i.e. the values that would be measured in case of uniform density;
(iv) more than 90% of counter-rotations start after the individual passes close to the centroid. A similar percentage indicates the counter-rotations that end with the passage close to the centroid of the individual; and
(v) in just over half of the counter-rotations the repulsions contribute significantly to the start or end (or both) of the individual's rotation in the direction opposite to that of the school of fish.

The last three points confirm and provide numerical consistency to the observations made in the previous subsections, using a few simulations as examples, regarding the non-uniformity of the milling school and the mechanisms for starting and ending the counter-rotation. Furthermore, the last point confirms that repulsions, although an important mechanism, are not decisive for starting and ending the counter-rotation.

### 3.4. Sensitivity to number of individuals and field of perception

Previous studies [17,42,47,48] have highlighted the importance of the number of individuals and the field of perception in relation to the states that the school of fish can reach and the spontaneous switches between different states that the school assumes during a given simulation. While the stability of the milling state increases with group size [42], the milling state does not occur for arbitrarily large groups [17]. More specifically, in [47] and [48] it is shown that, for certain ranges of the field of perception, increasing the number of individuals moves from a prevalence of the polar state to a prevalence of the milling state. Increasing the number further, the rotation coefficient decreases, making the transitional state prevail. In [48], it is also shown that while a perception field with almost no blind zone (i.e. *θ* close to 360°) favours the swarm state, reduction of the perception field favours the appearance, first, of the milling state, and after further reduction, of the polar state. This subsection analyses how the characteristics of counter-rotations change when *N* and *θ* change, i.e. when the states that the school of fish reaches change, maintaining the milling state for longer or shorter time intervals.

**Figure 7.**
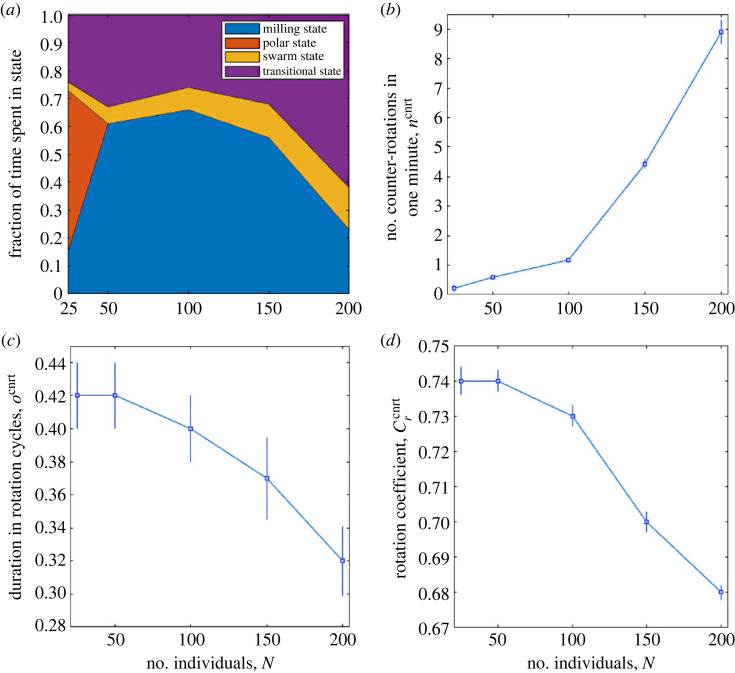
Collective behaviour and counter-rotation characteristics as a function of the number of individuals, *N*. For each *N*, 100 simulations, 2500 time steps each, were carried out. (*a*) Fraction of time spent by the school of fish in the four possible states. Sample mean (square dot) and s.e.m. (error bar) for: (*b*) *n*^cnrt^, (*c*) *o*^cnrt^, (*d*) $C_{r}^{\mathrm{cnrt}}$.

First, keeping the parameter values given in table 1 but making *N* take the values 25, 50, 100, 150, 200 and running 100 simulations for each value, the changes in collective behaviour and counter-rotation characteristics included in table 3 were observed. The profiles of greatest interest are shown in figure 7.

Figure 7*a* shows that although for *N* = 25 the polar state prevails, it rapidly disappears as the number of individuals increases, leaving room for the milling state. Then, increasing from 150 to 200 individuals, the milling state decreases considerably in favour of the transitional state. Recalling that *θ* = 270°, this behaviour is in line with observations in [17,42,47,48]. The swarm state is owing to the initial conditions imposed on the school of fish, and the time required to leave it increases moderately with *N*.

The number of counter-rotations occurring within 1 min increases significantly with *N*, as shown in figure 7*b*: moving from 25 to 200 individuals, the mean number of counter-rotations increases by about 45 times. The variability of the number of counter-rotations detected across simulations also grows considerably. Figure 7*c* and *d* show that, while the number of counter-rotations increases with *N*, their duration decreases moderately, from a mean of 0.42 rotation cycles to a mean of 0.32 rotation cycles (always maintaining high variability), and the rotation coefficient, measured during counter-rotations, also decreases slightly, indicating a decreasing uniformity of the milling school as *N* increases.

Second, keeping the parameter values given in table 1 (in particular, *N* = 100), the field of perception was varied. Preliminarily, it was verified that, for *θ* = 180°, a considerable fraction of time is spent in the polar state (about 35%), while the milling state never appears. At the opposite extreme, in the absence of the blind zone (i.e. *θ* = 360°), the polar and milling states occur for negligible fractions of time, while the school of fish spends its time in the swarm and transitional states. These outcomes are in accordance with the results in [48]. However, having to focus on counter-rotations, the field of perception values (discretized at 5° steps) used for further investigation are limited to those for which the milling state occurs for a fraction of time greater than 5%, i.e. the interval between *θ* = 225° and *θ* = 295°.

**Figure 8.**
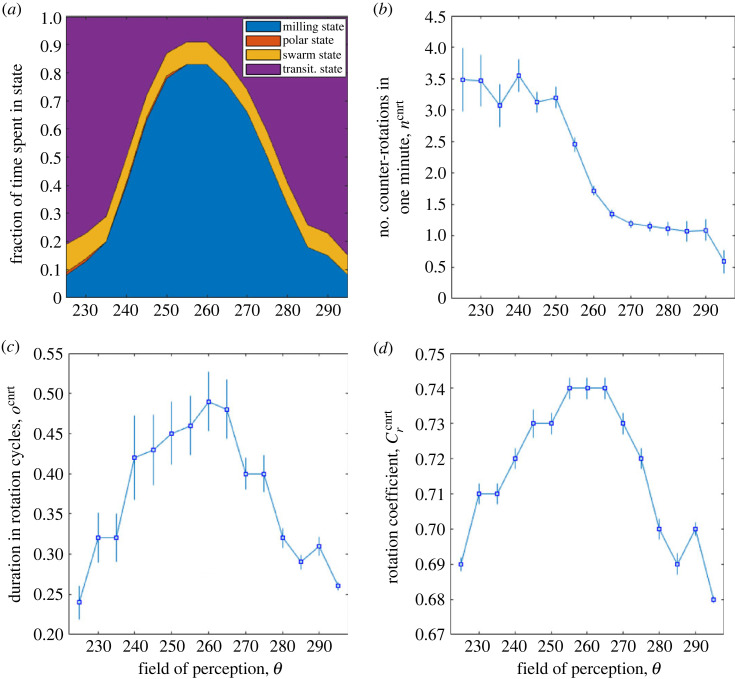
Collective behaviour and counter-rotation characteristics as a function of the field of perception, *θ*. For each *θ*, 100 simulations, 2500 time steps each, were performed. (*a*) Fraction of time spent by the school of fish in the four possible states. Sample mean (square dot) and s.e.m. (error bar) for: (*b*) *n*^cnrt^, (*c*) *o*^cnrt^, (*d*) $C_{r}^{\mathrm{cnrt}}$.

Figure 8 shows the results obtained performing 100 simulations for each value of *θ*. Figure 8*a* shows that the school of fish spends its time mainly in the milling and transitional states, with the proportion depending strongly on *θ*. The polar state is negligible and the swarm state, between 7% and 10%, represents the time the school needs to leave the state it is in at the beginning of each simulation, owing to random positions and orientations of fish. The number of counter-rotations per minute of milling state (figure 8*b*) is around unity for field of perception between 265° and 290°. When *θ* decreases (moving in the direction where the milling state disappears and is replaced by the polar state), the number rises significantly to about 3.5 counter-rotations min^−1^. The estimated s.e.m. also increases considerably. This increase in the mean and variability of counter-rotation number will be discussed in the next paragraph. Regarding the duration of counter-rotations (figure 8*c*) and the rotation coefficient at which they occur (figure 8*d*), these two quantities show the same profile, as already observed in figure 7. Such a profile is similar to the profile of the time the school of fish spends in the milling state. It can be argued that a longer stay of the school in the milling state is related to a higher value of the rotation coefficient (the sample mean of which, however, does not exceed 0.74, indicating poor uniformity within the torus), which in turn gives rise to counter-rotations of slightly longer duration (ranging from quarter to half a rotation cycle, with high variability).

**Figure 9.**
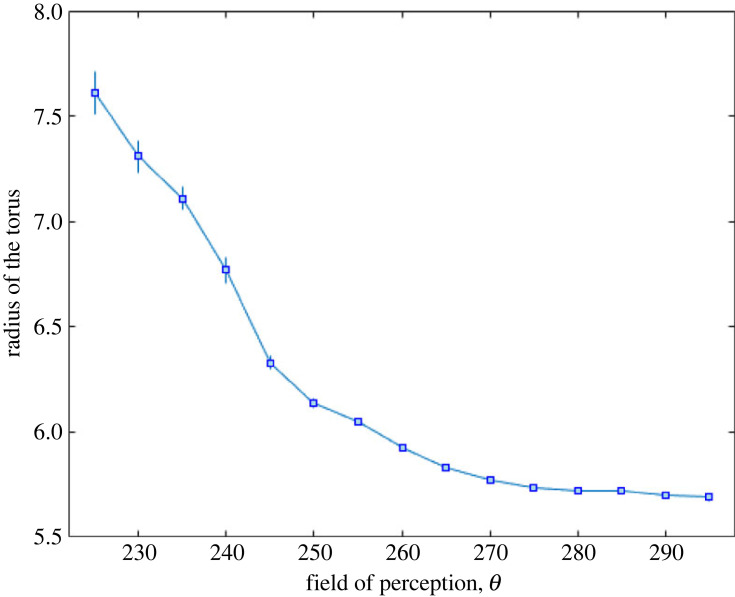
Sample mean (square dot) and s.e.m. (error bar) for the radius of the torus as a function of the field of perception. For each simulation, *R*_tor_(*t*) was calculated at each *t* at which the milling state is verified and averaged temporally.

The growth of sample mean and s.e.m. of the number of counter-rotations per minute, as the field of perception decreases, was investigated further. As the field of perception decreases, an individual in the rotating school is attracted less and less to fish located behind or to the side of it. As a result, the torus has more and more difficulty in staying closed. The fact that the percentage of time spent in this state decreases rapidly (figure 8*a*) as *θ* decreases confirms this fact. Further confirmation can be found by examining the radius of the torus as a function of the field of perception, shown in figure 9. By reducing the field of perception, the tori that are formed not only have shorter temporal durations but also exhibit an increasingly larger radius, on average, with greater variability. The increase in counter-rotations per minute as *θ* decreases thus seems related to the increased torus radius and the increasing difficulty in keeping the torus closed.

In simulations with 225° ≤ *θ* ≤ 245°, there are numerous instances where the school of fish reaches the milling state but, at a given instant, the torus is disrupted and the school moves to the transitional state. It can be speculated that this state transition is caused, to some extent, by counter-rotations, which, for such *θ* values, occur in large numbers. However, this assumption is not confirmed by the simulations, as the counter-rotations are distributed quite uniformly along the time interval when the school of fish is in the milling state, and not concentrated in the latter part of it, when the school approaches the transition. Therefore, the only conclusion supported by numerical evidence is that, for the set-up tested in this study, counter-rotations occur more frequently in tori whose large radius and short temporal duration are owing to the limited field of perception.

### 3.5. Counter-rotation in the presence of attraction only

On the one hand, it was verified in the previous subsections that repulsion is not decisive for starting and terminating the counter-rotation of an individual. On the other hand, the orientation interaction has been questioned several times because no evidence of it has been found for certain species of animals. For these reasons, it is of interest to perform tests in which the only interaction present between the individuals in the school of fish is what remains, i.e. attraction. As already documented in [23,26], attraction alone, together with asynchronous updating, is able to emerge milling, swarm and polar motion, depending on the values assigned to the parameters. Having fixed the number of individuals, *N*, and their speed, *v*_0_, the parameters that have the greatest impact on emergent behaviour turn out to be the field of perception, θ, and the maximum turning rate, *ρ*_max_. Furthermore, where the school of fish milling is the behaviour that emerges with high probability, depending on the values of the parameters, either double milling or single milling may prevail.

**Figure 10.**
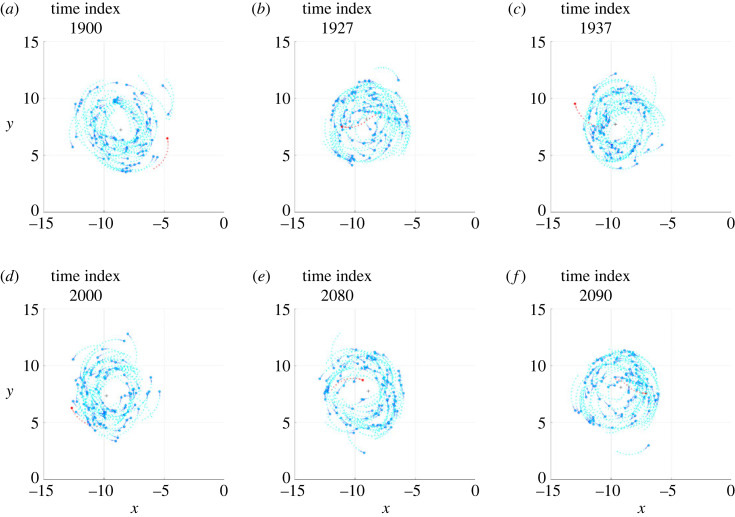
Six frames taken from the Sim*ε* simulation, the meaning of which is described in figure 1 caption, as well as the graphic conventions adopted.

**Figure 11.**
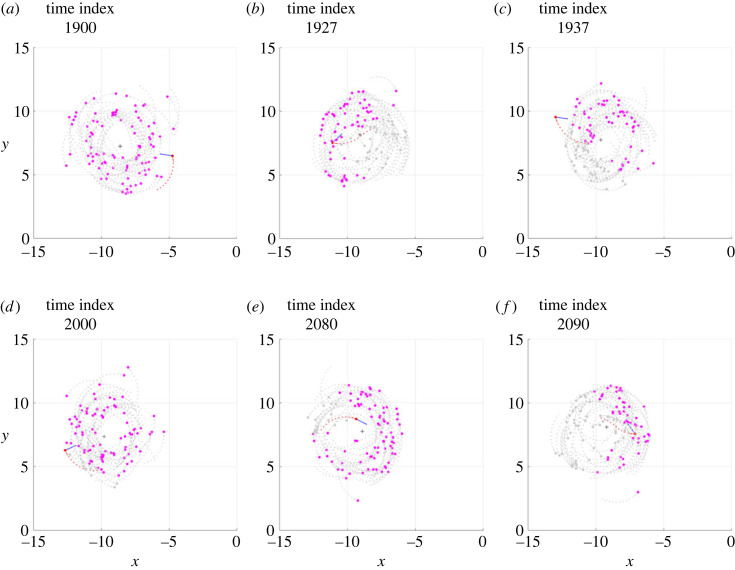
Six frames from the Sim*ε* simulation, the meaning of which is described in figure 1 caption. In this analysis the individual performing a temporary counter-rotation is in red; a blue vector of constant modulus is applied to it, indicating the direction of the vector $d_{\mathrm{des}}(t+\Delta_{t})$; the pink circles are the individuals exerting attraction, contained in the set $S_{a}$; the grey circles are the individuals exerting no interaction.

The values in table 2 were selected as capable of producing school of fish milling in which almost all individuals mill in the same direction. The absence of the orientation interaction, however, means that some individuals may mill in the opposite direction of the school in a stable manner, throughout the entire period in which the milling of the school is maintained. Neglecting individuals milling in the opposite direction of the school in a stable manner, it was possible to observe temporary counter-rotations, similar to those described in the previous subsections. The electronic supplementary material, videos S11 and S12 show, in three-dimensional space and in the *z* = 0 plane, respectively, a clip of a simulation, indicated with Sim*ε*, in which several individuals (not only the one highlighted in red) can be seen starting and ending the counter-rotation. With attraction alone, the phenomenon of temporary counter-rotation occurs more frequently than previously observed (as the quantitative analysis at the end of this subsection demonstrates), when repulsion and orientation interactions were also present. An analysis of the current interactions and the resulting desired direction can be seen in the electronic supplementary material, video S13. From the S12 and S13 videos, frames of the six salient phases, the same as those already identified for Sim*α* and Sim*β*, were extracted and shown in figures 10 and 11: milling in the same direction as the school of fish (*phase a*, figures 10*a* and 11*a*); passing close to the centroid and crossing the trajectories of other fish, moving outside the torus (*phase b*, figures 10*b* and 11*b*); beginning of milling in the opposite direction to the other fish (*phase c*, figures 10*c* and 11*c*); counter-rotation within the torus (*phase d*, figures 10*d* and 11*d*); the individual heads towards the centroid of the school (*phase e*, figures 10*e* and 11*e*); and passing close to the centroid and resuming milling in the same direction as the school of fish (*phase f*, figures 10*f* and 11*f*).

It can be seen that at the beginning of the counter-rotation, when the individual passes close to the centroid and moves outside the torus (*phase b*), it finds greater density in the fish that follow it (in the direction of the school of fish milling) than in those that precede it. The consequence, visible in figure 11*b*, is that the desired direction is turned backwards (i.e. in the opposite direction to that of the school of fish milling) gradually causing the individual to turn backwards and initiating its counter-rotation. In the Sim*α* and Sim*β* simulations, this result was produced by a combination of repulsions and different density between following and preceding fish (e.g. see what happens in *phase b* of Sim*β*, illustrated in figures 2*b* and 4*b*). In the present case, on the other hand, the reversal of the direction of milling is produced only by the difference in spatial density between the fish following the individual under consideration and those preceding it.

A similar phenomenon occurs to allow the individual to resume milling in the direction of the school of fish: the individual must move towards the centroid (*phase e*) and, as it moves towards the outside of the torus, it must find more density in the fish that precede it (in the direction of the school of fish milling) than in those that follow it (*phase f*). In this way will it be able to turn in the direction of the school milling (figure 11*f*), causing the individual to move forward and resume synchronous milling with the school of fish.

In analogy to previous subsections, the simulations described above are a few examples from 100 simulations performed with the parameter values listed in table 2. The analysis of all simulations reveals that the school of fish spends: 35% ± 3.4% of the time in the milling state; 13% ± 2.6% of the time in the swarm state; and 52% ± 3.2% of the time in the transitional state. The polar state is assumed only in negligible intervals. These numbers show the great variability in how long the polar, milling and transitional states are maintained in the different simulations and indicate a lower presence of the milling state than in the case of table 1.

Focusing on the time steps in which the school of fish is in the milling state, the sample mean and s.e.m. of the radius are 2.67 ± 0.02 units and those of the rotation period are 6.28 ± 0.02 s, showing that tori generated in these simulations, with only attraction, are significatively smaller than those obtained with the values in table 1.

The main characteristics of the counter-rotations observed in the 100 simulations are summarized in table 4. To avoid counting individuals that rotate in the opposite direction to the school of fish stably, counter-rotations are only considered if their duration is less than 80% of the time interval that the school spends in the milling state. Comparing the values in table 4 with those in table 3, one finds confirmation of what was anticipated above, describing the examples: the number of counter-rotations per minute increases greatly and is highly variable across simulations; the duration increases significantly and is, on average, about two cycles of rotation, with great variability; the passage of the individual near the centroid, in the starting and ending phases, characterizes the majority of counter-rotations.

**Table 4.** Characteristics of the counter-rotations observed in 100 simulations with the parameter settings of table 2.

counter-rotation characteristic	symbol	value
number of occurrences per minute	*n*^cnrt^	6.77 ± 1.01
duration in rotation cycles	*o*^cnrt^	1.95 ± 0.35
polarization coefficient	$C_{p}^{\mathrm{cnrt}}$	0.25 ± 0.008
rotation coefficient	$C_{r}^{\mathrm{cnrt}}$	0.74 ± 0.005
passage near the centroid (start)	*P*_1_	62.2%
passage near the centroid (end)	*P*_2_	60.9%
significant repulsions (start OR end)	*P*_3_	not applicable

### 3.6. Importance of uniformity of fish distribution within the torus

The importance of the non-uniform distribution of fish within the torus for the starting of counter-rotations was emphasized in relation to both the cases of three active interactions and the case of attraction only. In this subsection, conditions that produce high uniformity of fish within the torus are set and the impact on counter-rotations is analysed. Instead of random initial positions and orientations, the fish are randomly arranged on the lateral surface of a right circular cylinder, with vertical axis, radius of 10 units and height of 4 units. Fish are oriented on horizontal lines, tangent to the cylinder, all pointing in the same direction of rotation. To maintain the high uniformity with which the fish are initially distributed, the three interactions are activated using the values listed in table 1, with two exceptions: a smaller repulsion sphere, *R_r_* = 0.2 unit; and a lower maximum turn rate, *ρ*_max_ = 20 degree s^−1^.

With the above set-up, the school of fish is already in the milling state at the start. Furthermore, in all the 100 simulations performed, the milling state is maintained for the entire duration (i.e. 2500 time steps). The uniformity in the fish distribution within the torus is proved by the rotation coefficient which, in all simulations, is always greater than 0.92 and by the polarization coefficient, always less than 0.18. Under these conditions, no counter-rotation was detected in any of the simulations performed.

If *R_r_* = 0.2 unit and *ρ*_max_ = 20 degree s^−1^ are kept, but random initial positions inside a sphere with radius Y = 10 and random initial orientations are reintroduced, 100 simulations show that the school of fish spends 60.4% of the time steps in the milling state and 2.98 ± 0.30 counter-rotations min^−1^ are observed. The sample mean and s.e.m. of the rotation coefficient during these counter-rotations are 0.75 ± 0.006, indicating poor uniformity. This confirms that uniformity in the distribution of individuals within the torus is the key factor in the presence (or absence) of counter-rotations.

## 4. Discussion

First of all, it should be recalled that the simulations described in this paper have distinctive elements, which are uncommon in other studies on collective motion models: the individuals in the school of fish can move in a three-dimensional space; the initial conditions of each of them (i.e. position and orientation at the first time instant) are set randomly; and the update between one time step and the next occurs asynchronously.

With regard to the starting of counter-rotation, the results described in the previous section allow one to deduce two key aspects illustrated below, both of which appear to be confirmed by the visual analysis of the underwater video recordings described in the link list contained in the electronic supplementary material:

(i) the individual entering counter-rotation comes from inside the torus and moves to the outside of the torus, following a path that crosses the trajectories of other milling individuals; and
(ii) the fact that the individual exiting the torus orients itself in the opposite direction to the school of fish may be owing to: the strong attraction of the fish following it (in the direction of the school milling), since they belong to a region with a greater density than the density of the fish preceding it; repulsions occurring during the passage from the inside of the torus to the outside; or a combination of these two mechanisms.

Subsequently, the orientation interaction does not prevent the individual from milling in the opposite direction. This is possible because, with the values set for the parameters, the attraction interaction is dominant. On ending the counter-rotation, something similar to the starting phase must occur, i.e. (i) the individual moves to the inner part of the torus, (ii) from the inner part the individual moves outwards, (iii) the individual changes direction of milling thanks to: the attraction of those in front of it (since they belong to a region with greater density); the action of repulsions; or the combination of the two previous mechanisms.

Finally, it should be noted that the counter-rotation can be of very variable duration, ranging from a short arc of circumference to several turns. The counter-rotation will last until the individual falls in the conditions described above, those that allow it to resume milling in the direction of the school of fish. This fact also corresponds to what can be seen in the underwater video recordings described in the link list contained in the electronic supplementary material.

## 5. Conclusion

Studies based on the well-known three-zone model have shown that, in addition to the emergence of a clear collective behaviour, when the school of fish undertakes a milling motion, this model does not prevent the occurrence of individual behaviour that disagrees with the collective one. The individual undertaking to mill in the opposite direction of the school and subsequently returning to mill in the same direction as the school (i.e. temporary counter-rotation) is an event observed quite commonly in three-dimensional simulations in which the school of fish goes into milling. These simulations start with random initial conditions and proceed with asynchronous updated, including repulsion, orientation and attraction interactions. The temporal density of counter-rotations increases with the number of individuals in the school and as their field of perception decreases (i.e. going in the direction in which the milling state gives way to the polar state). By reducing the field of perception, the school of fish remains in the milling state for less time. Although the increase in counter-rotations accompanies the decreased stability of the torus (owing to the reduced perceptual field), there is no evidence that counter-rotations are the cause of torus disruption.

A detailed analysis of some examples of temporary counter-rotation has shown that it originates from inhomogeneities in the density of the fish present along the circumference and from the repulsions experienced by individuals who, being inside the torus, follow a trajectory that leads them outside the torus. For the same reasons, to which the contribution of the orientation interaction can be added, the counter-rotation is subsequently terminated. It has also been observed that the contribution of repulsion is not decisive: counter-rotation can begin and end without the help of repulsion. Indeed, even if only the attraction interaction is activated, for specific parameter values, the model allows the milling of the school of fish to emerge and individual temporary counter-rotations to be observed. The validity of these observations is confirmed by numerical investigations performed through objective analysis of many independent simulations. The numerical assessment has also shown that counter-rotations do not occur when fish are distributed within the torus with high uniformity.

Some underwater video recordings of milling schools of fish show that counter-rotation of an individual is a real phenomenon and that it apparently originates for similar reasons to those observed in simulations. This does not mean that the versions of the three-zone model used in this paper can explain the real reasons why some fish mill, over a period of time, in the opposite direction to the school to which they belong. More simply, this paper has helped to draw attention to a little-investigated phenomenon, showing how certain models that are already widely used generate, under appropriate conditions, not only dominant collective behaviour but also individual behaviour that is discordant with that of the group. The interactions and mechanisms that make this possible in the simulation can guide the ethological study of individual fish behaviour without any claim to anticipate its conclusions.

This paper addresses the case of constant speed motion and choice of interacting neighbours based on the geometric distance from the individual under consideration. Future research activities could investigate the presence and the characteristics of counter-rotations when different motion models (in particular, the burst-and-coast type of swimming) and topological schemes for the selection of interacting neighbours are adopted, possibly combining these two aspects [51].

## Figures and Tables

**Figure 12.**
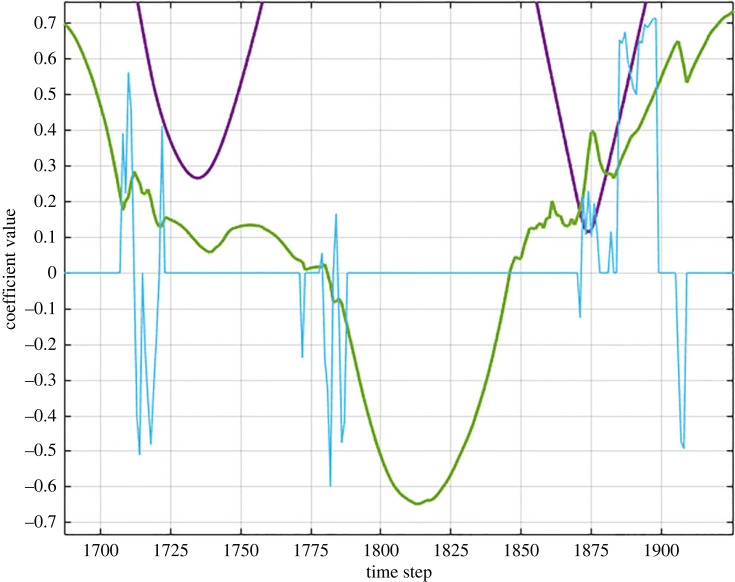
Example of counter-rotation of a given fish. Green line: counter-rotation coefficient, *c_i_*(*t*). Purple line: $\left|r_{i}(t)-r_{c}(t)|/R_{\mathrm{tor}}(t\right)$. Light blue line: repulsion coefficient, *p_i_*(*t*). See the text for further details.

## Data Availability

The datasetes and Matlab code supporting this paper have been uploaded as part of the electronic supplementary material (see Data_and_Code.zip) [52].

## Appendix A

In this section, the algorithm for calculating the percentages *P*_1_, *P*_2_ and *P*_3_, introduced in §3.3 and used in tables 3 and 4, is described with reference to the example shown in figure 12. The green line shows the counter-rotation coefficient, *c_i_*(*t*), of an individual *i* that performs a counter-rotation. The coefficient *c_i_*(*t*) is less than −0.3 in the time steps between 1793 and 1837: these two bounds are denoted by *t*_1_ and *t*_2_, respectively, and indicate the start and end of the counter-rotation. (Here, the variable *t* is assumed to represent the time step index.) The purple line shows the value of: $\left|r_{i}(t)-r_{c}(t)|/R_{\mathrm{tor}}(t\right)$, i.e. the distance of the *i*-th individual from the centroid with respect to the torus radius. One can see that, before the counter-rotation start and after its end, this variable shows two local minima, located in *t_α_* and *t_β_* (in the example: *t_α_* = 1735 and *t_β_* = 1874), with amplitudes *m_α_* and *m_β_*, respectively. These minima indicate the passage of the *i*-th individual close to the centroid. The light blue line shows the repulsion coefficient, *p_i_*(*t*), for the *i*-th individual. The negative values assumed by *p_i_*(*t*) between *t_α_* and *t*_1_ indicate the repulsions that contributed to start the rotation in the direction opposite to that of the school. On the contrary, the positive values assumed by *p_i_*(*t*) after *t_β_* indicate the repulsions that contributed to resume the rotation in the same direction as the school.

The percentage *P*_1_ measures the counter-rotations for which, in the starting phase, the individual passes near the centroid. Referring to the notation introduced above, a given counter-rotation contributes to the percentage *P*_1_ if the following condition is met: the minimum value of $\left|r_{i}(t)-r_{c}(t)|/R_{\mathrm{tor}}(t\right)$ in [*t*_1_ − 50, *t*_1_], called *m_α_* and located in *t_α_*, is less than or equal to 0.5. This means that the distance of the *i*-th individual from the centroid is considered over an interval of 50 time steps before the counter-rotation starts.

The percentage *P*_2_ measures the counter-rotations for which, in the ending phase, the individual passes near the centroid. Similarly to above, a given counter-rotation contributes to the percentage *P*_2_ if the following condition is met: the minimum value of $\left|r_{i}(t)-r_{c}(t)|/R_{\mathrm{tor}}(t\right)$ in [*t*_2_, *t*_2_ + 50], called *m_β_* and located in *t_β_*, is less than or equal to 0.5.

Focusing on the counter-rotation of the *i*-th individual, the repulsions it undergoes are considered significant for starting the counter-rotation if the following condition is met:

A.1 $$\sum_{t_{\alpha }}^{t_{1}}\;p_{i}(t)<-1.$$

Recalling that *p_i_*(*t*) is for constructions between −1 and 1, to satisfy this condition, it is necessary that, between the passage near the centroid and the start of counter-rotation, there are multiple repulsions that push the fish to rotate in the direction opposite to that of the school. Analogously, the repulsions the *i*-th individual undergoes are considered significant for ending the counter-rotation if the following condition is met:

A.2 $$\sum_{t_{\beta }}^{t_{\beta }+20}\;p_{i}(t)>1.$$

To satisfy this condition, it is necessary that, in the 20 time steps after the passage near the centroid, there are multiple repulsions that push the fish to rotate in the same direction as that of the school. If at least one of the two conditions above is met, then the counter-rotation under examination contributes to the percentage *P*_3_.
